# Supplementary material for: Impact of Tumor Size on Prognosis in Differentiated Thyroid Cancer with Gross Extrathyroidal Extension to Strap Muscles: Redefining T3b
Source: Cancers (Basel). 2024 Jul 18;16(14):2577. doi: 10.3390/cancers16142577 (PMC11274482; doi:10.3390/cancers16142577)
Supplement: Supplementary file 1 [file cancers-16-02577-s001.zip › cancers-3050018-supplementary.pdf]

Supplementary Table S1. Comparisons of baseline characteristics between T categories and T3b subcategories within the same size range (T1 vs. T3b-1, T2 vs. T3b-2, and T3a vs. T3b-3).

	T1 (n = 5535)	T3b-1 (n = 239)	*p*-value	T2 (n = 339)	T3b-2 (n = 90)	*p*-value	T3a (n = 59)	T3b-3 (n = 20)	*p*-value
**Age (years)**	46.6 ± 12.0 (range, 12–88)	49.6 ± 12.6 (range, 11–81)	<0.001	44.0 ± 14.7 (range, 12–83)	52.5 ± 15.7 (range, 14–83)	<0.001	46.4 ± 15.1 (range, 16–77)	49.8 ± 16.4 (range, 18–81)	0.403
**Female**	4437 (80.2%)	202 (84.5%)	0.097	222 (65.5%)	62 (68.9%)	0.544	37 (62.7%)	11 (55.0%)	0.542
**Extent of surgery**			<0.001			<0.001			0.004
Lobectomy	3442 (62.2%)	215 (90.0%)		253 (74.6%)	89 (98.9%)		36 (61.0%)	19 (95.0%)	
TT and/or mRND	2093 (37.8%)	24 (10.0%)		86 (25.40%)	1 (1.1%)		23 (39.0%)	1 (5.0%)	
**Aggressiveness** (Aggressive variant PTC, Widely invasive FTC)	226/5522 (4.1%)	16/238 (6.7%)	0.048	27/331 (8.2%)	12/88 (13.6%)	0.116	9/57 (15.8%)	2/18 (11.1%)	1.000
**Tumor size (cm)**	0.8 ± 0.4 (range, 0.01–2.0)	1.2 ± 0.4 (range, 0.2–2.0)	<0.001	2.8 ± 0.5 (range, 2.1–4.0)	2.8 ± 0.5 (range, 2.1–4.0)	0.582	5.3 ± 1.5 (range, 4.1–13.5)	5.5 ± 1.3 (range, 4.2–9.0)	0.707
**Multifocality**	1984 (35.9%)	119 (49.8%)	<0.001	124 (36.6%)	47 (52.2%)	0.005	25 (42.4%)	7 (35.0%)	0.562
**Lymphatic invasion**	1304/5183 (25.2%)	123/226 (54.4%)	<0.001	135/327 (41.3%)	64/88 (72.7%)	<0.001	17/56 (30.4%)	18/18 (100.0%)	<0.001
**Vascular invasion**	93/5133 (1.8%)	15/221 (6.8%)	<0.001	38/327 (11.6%)	8/86 (9.3%)	0.543	15/57 (26.3%)	7/19 (36.8%)	0.381
**$BRAF^{V600E}$ positivity**	3679/4544 (81.0%)	163/193 (84.5%)	0.225	167/262 (63.7%)	60/67 (89.6 %)	<0.001	20/39 (51.3%)	12/13 (92.3%)	0.008
**Harvested LNs**	11.5 ± 13.5 (range, 0–168)	20.2 ± 23.7 (range, 1–185)	<0.001	21.2 ± 23.6 (range, 1–122)	31.1 ± 25.2 (range, 1–101)	0.001	29.9 ± 37.9 (range, 0–132)	52.7 ± 37.3 (range, 1–134)	0.026
**Positive LNs**	1.9 ± 3.8 (range, 0–74)	4.3 ± 5.7 (range, 0–29)	<0.001	5.0 ± 7.1 (range, 0–41)	7.4 ± 7.2 (range, 0–36)	0.006	7.8 ± 15.0 (range, 0–71)	15.8 ± 13.8 (range, 0–46)	0.045
**N stage**			<0.001			<0.001			0.001
N0 + Nx	3114 (56.3%)	72 (30.1%)		158 (46.6%)	16(17.8%)		30 (50.9%)	2 (10.0%)	
N1a	2038 (36.8%)	119 (49.8%)		108 (31.9%)	31(34.4%)		11 (18.6%)	2 (10.0%)	
N1b	383 (6.9%)	48 (20.1%)		73 (21.5%)	43(47.8%)		18 (30.5%)	16 (80.0%)	
**TNM stage**			<0.001			<0.001			0.238
Stage I	5016 (90.6%)	148 (61.9%)		303 (89.4%)	47 (52.2%)		41 (69.5%)	11 (55.0%)	
Stage II	519 (9.4%)	91 (38.1%)		36 (10.6%)	43 (47.8%)		18 (30.5%)	9 (45.0%)	
**Recurrence**	156 (2.8%)	16 (6.7%)	0.001	35 (10.3%)	20(22.2%)	0.003	8 (13.6%)	7 (35.0%)	0.049
**Overall mortality**	101(1.8%)	6 (2.5%)	0.455	12 (3.5%)	10 (11.1%)	0.012	4 (6.8%)	6 (30.0%)	0.014
**Disease specific mortality**	8 (0.1%)	1 (0.4%)	0.317	2 (0.6%)	6 (6.7%)	0.001	1 (1.7%)	5 (25.0%)	0.003

Data are expressed as patient's number (%), or mean ± SD. A statistically significant difference was defined as $p < 0.05$. Abbreviations: TT, total thyroidectomy; mRND, modified radical neck dissection; PTC, papillary thyroid carcinoma; FTC, follicular thyroid carcinoma; LN, lymph node; T, tumor; N, node; M, metastasis.
